# Hepatic Encephalopathy Confirmed by Magnetic Resonance Imaging in a Patient with Unobvious Cause of Chronic Liver Disease Decompensation

**DOI:** 10.3390/diagnostics13040753

**Published:** 2023-02-16

**Authors:** Piotr F. Czempik, Michał P. Pluta, Mariusz Hofman, Piotr S. Liberski, Tomasz Jaworski, Anna Szczepańska, Oskar Bożek

**Affiliations:** 1 Department of Anaesthesiology and Intensive Care, Faculty of Medical Sciences in Katowice, Medical University of Silesia, 40-752 Katowice, Poland; 2 Department of Radiodiagnostics and Invasive Radiology, Faculty of Medical Sciences in Katowice, Medical University of Silesia, 40-752 Katowice, Poland

**Keywords:** acute liver failure, chronic liver disease, cirrhosis, encephalopathy, magnetic resonance imaging

## Abstract

Fifty-four-year old male was admitted to the intensive care unit (ICU) due to impaired consciousness. Past medical history included alcohol dependence, liver cirrhosis, esophageal varices, 2 esophageal varices banding procedures in the past, pathological obesity. Computed tomography (CT) examination of the head performed in the referring hospital was normal. At admission the CT examination of the head was repeated and showed no abnormalities. Urgent esophagogastroduodenoscopy revealed presence of esophageal varices and scarification following previous banding procedures located in the middle and lower part of the esophagus. Gastrointestinal bleeding being the most likely cause of chronic liver decompensation was therefore excluded. Multimodal neurologic diagnostic assessment was negative. Finally magnetic resonance imaging (MRI) of the head was performed. Taking into account clinical picture and the MRI result, the differential diagnosis included chronic liver encephalopathy, exacerbated acquired hepatocerebral degeneration, and acute liver encephalopathy. Due to history of umbilical hernia CT of the abdomen and pelvis was performed and showed intussusception of the ileum, confirming hepatic encephalopathy. In this case report the MRI suggested the diagnosis of hepatic encephalopathy and prompted search for alternative causes of decompensation of chronic liver disease.

## 1. Case Presentation

Fifty-four-year old male was admitted to the intensive care unit (ICU) due to impaired consciousness (Glasgow Coma Scale score 6) and acute respiratory failure. Recently the patient complained of malaise, drowsiness, problems with memory (was not able remember names of known objects), visual hallucinations (saw ‘flowers’). The patient in the last 3 days experienced several episodes of non-bloody vomiting and constipation. Past medical history included: alcohol dependence—the patient ceased to consume alcohol after the last esophageal varices banding procedure approximately 5 months earlier, liver cirrhosis (Child-Pugh class B), esophageal varices (grade II/III), 2 esophageal varices banding procedures, secondary thrombocytopenia, cholelithiasis, pathological obesity, epilepsy. Computed tomography (CT) examination of the head performed in the referring hospital was normal. At admission to the ICU the head CT examination was repeated and showed no abnormalities. The physical examination of the abdomen did not reveal any pathologic finding, however its diagnostic value was limited because of obesity. 

## 2. Differential Diagnosis of Impaired Consciousness

**Figure 1:**
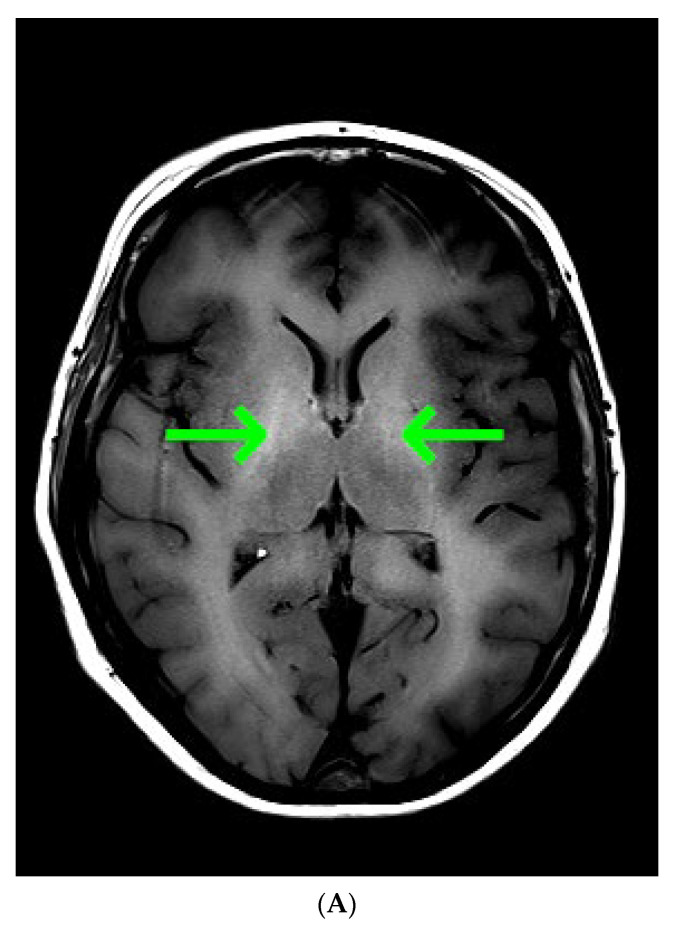

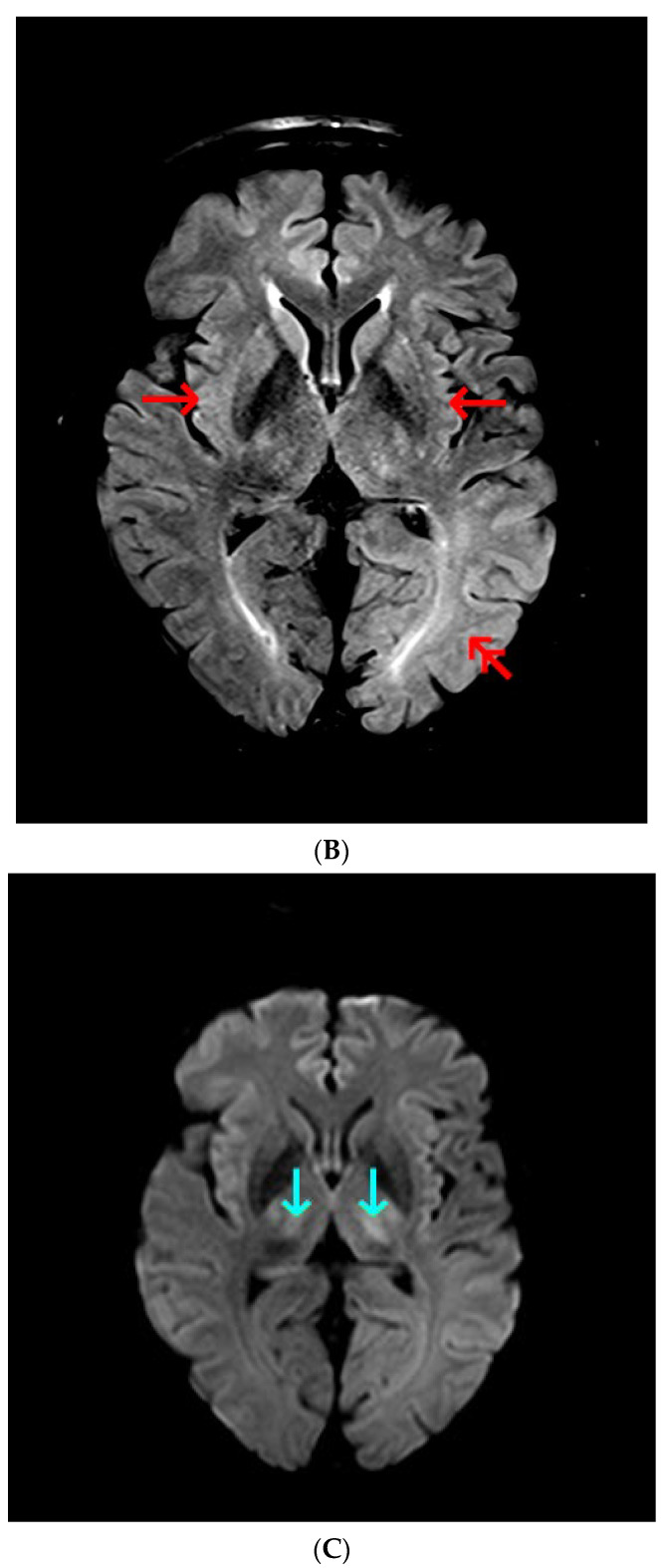

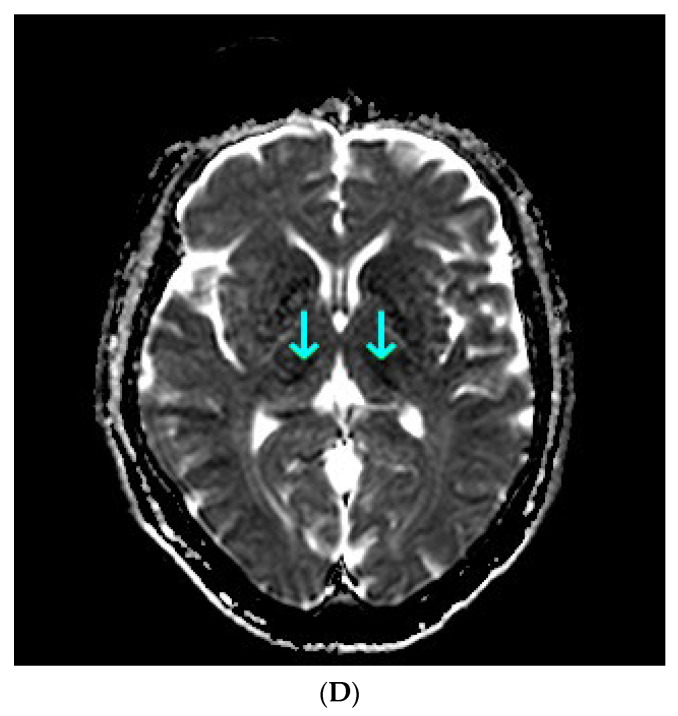
(**A**) Magnetic resonance imaging of the head. T1 Spin Echo, axial. Green arrows point to hyper-intense globi pallidi; (**B**) Magnetic resonance imaging of the head. T2 Turbo Spin Echo Dark-fluid. Red single arrows point to the mild symmetric hyper-intensity in the insulas; double red arrow point to the pronounced hyper-intensity in the left occipital lobe; (**C**) Magnetic resonance imaging of the head. Diffusion Weighted Imaging b = 1000, axial. Cyan arrows point to the restricted water diffusion in both thalami; (**D)** Magnetic resonance imaging of the head. Apparent Diffusion Coefficient, b = 0, b = 1000, axial. Cyan arrows point to the restricted water diffusion in both thalami.

Common causes of decompensation of chronic liver disease include gastrointestinal (GI) bleeding, infection, high alcohol intake and hepatotoxic medications [1]. As the last three were unlikely, urgent esophagogastroduodenoscopy was ordered, however it showed non-bleeding esophageal varices and scarification following previous banding procedures in the middle and lower part of the esophagus. Gastrointestinal bleeding being the most likely cause of chronic liver decompensation was therefore excluded. In order to find the cause of impaired consciousness multimodal diagnostic assessment was initiated. Toxic screen showed absence of phenobarbital, carbamazepine, benzodiazepines, tricyclic antidepressants; valproic acid was present in the subtherapeutic concentration of 41 µg/mL (therapeutic range 50–100 µg/mL). On day 3 of ICU hospitalization angio-CT of the head was performed and also revealed no abnormalities. The EEG showed no signs of epileptiform activity, although generalized inhibition of basal activity was present. The direct chemiluminescence of the cerebrospinal fluid (CSF) performed on day 4 showed absence of IgG and IgM against B. burgdorferii, Herpes simplex virus, and cytomegalovirus. There was no DNA of Ebstein-Barr virus detected in the CSF sample. The onco- and anti-neuronal immunoglobulin panel performed with indirect immunofluorescence was negative: anty-Amp, anty-Cv2.1, anty-PNM2/Ta (Ma2/Ta), anty-Ri, anty-Yo, anty-Hu, anti-Rec, anti-SOX1, anti-titin. The anti-neuronal screen performed with indirect immunofluorescence was negative: anty-AMPA (GluR1/GluR2), anty-GABA B, anty-NMDA, anty-DPPX, anty-CASPR 2, anty-LGl 1). All initial microbiology cultures (blood, aspirate, urine, CSF) were negative. As part of diagnostic assessment the magnetic resonance imaging (MRI) examination of the head was performed on day 5. The image obtained by MRI is depicted in Figure 1A–D. 

Taking into account clinical picture the differential diagnosis involved: chronic liver encephalopathy, acquired hepatocerebral degeneration (AHD), acute liver encephalopathy. Various nonspecific, not limited to vascular supply region, lesions of the brain, including rare appearance like T1 gray matter hyper-intensity, were consistent with hepatic encephalopathy. Because liver encephalopathy was a possible diagnosis, ammonia concentration was determined. The ammonia concentration determined with spectrophotometry reached 417 µg/dL, highly above the reference range (31–123 µg/dL). Ammonia was found to be an independent risk factor for hepatic encephalopathy and intracranial hypertension, the latter occurring in 55% of patients with ammonia concentrations >200 μmol/L (340.6 µg/dL) [2].

**Figure 2:**
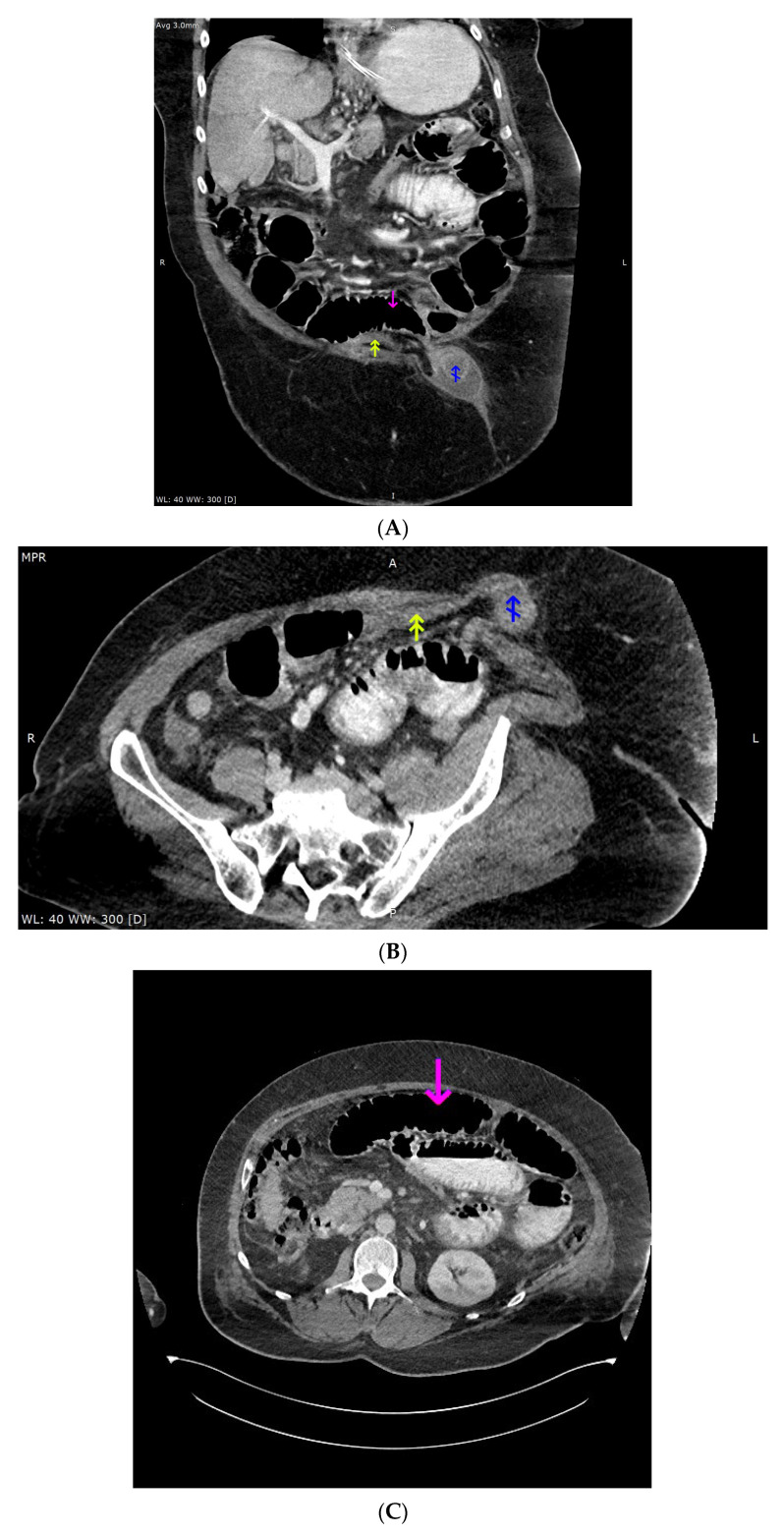
(**A**) Computed tomography imaging of the abdomen and pelvis, portal-venous phase, oral contrast administered, oblique (closest to coronal) reconstruction (3 mm). Magenta arrow points to the pre-stenotic, dilated small intestine, lumen mostly filled with gas; double yellow arrow points to the post-stenotic, small intestine without any content; blue arrow points to the edematous small intestine loop inside the hernia sac, surrounded by fluid; (**B**) Computed tomography imaging of the abdomen and pelvis, portal-venous phase, oral contrast administered, oblique (closest to axial) reconstruction (3 mm). Double yellow arrow points to the post-stenotic, small intestine without any content; blue arrow points to the edematous small intestine loop inside the hernia sac, surrounded by fluid. (**C**) Computed tomography imaging of the abdomen and pelvis, portal-venous phase, oral contrast administered, axial reconstruction (1.5 mm). Magenta arrow points to the pre-stenotic, dilated small intestine, lumen mostly filled with gas.

Due to history of umbilical hernia, computed tomography (CT) of the abdomen and pelvis was performed and showed radiographic signs of mechanical bowel obstruction with dilation of jejunum and ileum proximal to the hernia (Figure 2A–C).

The patient underwent emergency resection of the intussuscepted ileum. The intussusception of the small intestine was described previously a cause of acute-on-chronic liver failure [3,4]. Due to high ammonia concentration and status post intestinal surgery with potential for further ammonia increase, a series of single-pass albumin dialysis (SPAD) procedures was performed. The ammonia concentration decreased with successive SPAD procedures to 252, 100, and 98 µg/dL. The patient died from multi-organ failure. 

## 3. Conclusions

In this case report the MRI suggested the diagnosis of hepatic encephalopathy and prompted search for alternative causes of decompensation of chronic liver disease, whereas CT confirmed the cause of decompensation of the disease. 

## Data Availability

The data presented in this study are available on request from the corresponding author. The data are not publicly available due to privacy concerns.
